# Cytokines and chemokines measured in dried SLA-stimulated whole blood spots for asymptomatic *Leishmania infantum and Leishmania donovani* infection

**DOI:** 10.1038/s41598-017-17315-z

**Published:** 2017-12-08

**Authors:** A. V. Ibarra-Meneses, D. Mondal, J. Alvar, J. Moreno, E. Carrillo

**Affiliations:** 1 WHO Collaborating Centre for Leishmaniasis, Centro Nacional de Microbiología, Instituto de Salud Carlos III, Madrid, Spain; 2 Nutrition and Clinical Services Division (NCSD), International Centre for Diarrhoeal Disease Research, Dhaka, Bangladesh; 3 Drugs for Neglected Diseases Initiative (DNDi), Geneva, Switzerland

## Abstract

Whole blood stimulation with soluble *Leishmania* antigen (SLA), followed by plasma cytokine and chemokine determination, provides means of detecting subjects with asymptomatic *Leishmania* infection. This work examines the potential of Protein Saver 903 cards for the storage and transport of SLA-stimulated dried plasma spot samples. Blood was collected from asymptomatic and negative control subjects living in a *Leishmania infantum-* (Spain) and *Leishmania donovani*-endemic area (Bangladesh). After SLA-stimulation, three types of sample were prepared: frozen liquid plasma (−20 °C), and plasma dropped onto Protein Saver cards kept at −20 °C (DPS-FZ), and at ambient temperature (DPS-AT). The concentrations of IFN-γ, IL-2, CXCL10, CXCL9, CCL2 and CXCL8 in the thawed liquid plasma (TLP), DPS-FZ and DPS-AT samples were then determined. Strong correlations were seen between the TLP and DPS-FZ/AT samples for all the studied cytokines/chemokines in both the *L. infantum* and *L. donovani* areas. Protein Saver 903 cards would therefore appear to allow for the transport of SLA-stimulated plasma samples by courier at ambient temperature. The CXCL10 and CXCL9 detectable in these plasma spots provided robust markers for identifying asymptomatic subjects from both endemic areas. This easy procedure opens up new possibilities for field studies in resource-limited settings, which could help in *Leishmania* control.

## Introduction

Leishmaniasis is a chronic infectious disease caused by a group of protozoan parasites of the genus *Leishmania*. It can be fatal, yet it remains one of the most neglected of all tropical diseases^1^. Asymptomatic *Leishmania* infections are, however, more common than visceral leishmaniasis^2,3^, and simple, standardized, high-throughput screening tools are therefore needed not only to help control the disease where it is endemic, but to identify infected but asymptomatic subjects when recruiting for clinical trials^4,5^.

The stimulation of whole blood with soluble *Leishmania* antigen (SLA), a procedure employed to examine cell-mediated immunity against the parasite, is a rapid, stable, robust and sensitive field method particularly useful for detecting asymptomatic subjects^6–9^. However, the transport of SLA-stimulated plasma samples to analytical laboratories requires a cold chain be maintained. Safety is also a problem since such samples are potentially infectious^10^. A simpler, safer, yet robust method of transporting such samples from remote regions is needed.

Since 1963, Protein Saver cards (Whatman) have been used for storing and transporting neonate blood samples taken during screening programmes^11^. In recent years these cards have been used to transport dried plasma spot (DPS) and dried blood spot (DBS) samples from patients with HIV, *Mycobacterium Tuberculosis*, Cytomegalovirus and *Leishmania* infection^12–15^, although not for the detection of cytokines/chemokines (the focus of the present work).

Skogstrand *et al*. (2005) were the first to detect cytokines and chemokines from neonatal blood on filter paper-based systems using a multiplex assay^16^. A few studies have explored this avenue after whole blood stimulation for diagnosing bovine brucellosis and human tuberculosis^10,15,17–19^. However, such systems have not been tested with regard to the detection of cytokines and chemokines for the identification of *Leishmania* infection even though they potentially offer a safer and more logistically-friendly alternative to the transport of frozen SLA-stimulated plasma samples. They could, therefore, be of great use in *Leishmania* control programmes, validation studies, and clinical trials.

The aim of the present work was to determine whether plasma from SLA-stimulated blood transported on Protein Saver 903 cards at ambient temperature (DPS-AT) for 10 days can be used instead of liquid plasma samples (which have to be transported frozen) for the later detection of cytokines/chemokines - interferon gamma (IFN-γ), interleukin-2 (IL-2), IFN-γ-induced protein 10 (IP-10 or CXCL10), the monokine induced by IFN-γ (MIG or CXCL9), monocyte chemoattractant protein-1 (MCP-1 or CCL2) and interleukin-8 (IL-8 or CXCL8) - and thus the identification of subjects with asymptomatic *Leishmania* infection. The results show that CXCL10 and CXCL9 on Protein Saver 903 cards can serve as biomarkers of asymptomatic subjects even after a postal journey at ambient temperature. This could be a boon for *Leishmania* control in resource-limited settings.

## Materials and Methods

### Ethics statement

This study was approved by the *Hospital de Fuenlabrada* (Madrid) Ethics and Research Committee, and by the Ethical Review Committee of the International Centre for Diarrhoeal Disease Research, Bangladesh. All participants gave their written consent to be involved. All experiments were performed in accordance with current guidelines and regulations.

### Experiment 1. Proof of concept that Protein Saver cards can be used to transport SLA-stimulated dried plasma samples

Blood was collected from 40 asymptomatic subjects (as confirmed via a standard cell proliferation assay [CPA] performed following a previously described methodology^7^) plus 20 CPA-negative control subjects during their attendance at the Hospital de Fuenlabrada Blood Bank (Madrid, Spain) in 2016–2017. All subjects lived in a *L. infantum*-endemic area, close to the reference laboratory where all analyses were performed. All 60 samples were PCR-, direct agglutination (DAT)- and rK39- negative, as revealed following previously described methods^20^.

Fractions (500 µL) of these blood samples were left either untreated (control) or were SLA-stimulated for 24 h as previously described^6^. Plasma (collected from the now naturally separated blood fractions) was frozen at −20 °C until analysis 10 days later (a period designed to mimic cold-chain-maintained transport to the laboratory via courier). After thawing at ambient temperature and diluting 1/10 in PBS 1X, the IFN-γ, IL-2, CXCL10, CXCL9, CCL2 and CXCL8 concentrations were determined using the Cytometric Bead Array Human Soluble Protein Flex Set (Becton Dickinson, Franklin Lakes, NJ, USA), following the manufacturer’s instructions. Results for each cytokine and chemokine were expressed as the difference between the SLA-stimulated and control plasma concentrations.

Thawed liquid plasma (TLP) was also dropped onto two pre-marked 1.2 cm-diameter circles on separate Protein Saver 903 cards, and dried for 3–4 h at ambient temperature (AT) in a horizontal position on the bench to produce dried plasma spots (DPS). The cards were then placed in zip-lock plastic bags containing a desiccant and maintained at either −20 °C (DPS-FZ) or ambient temperature (DPS-AT) (again to mimic transport to the laboratory via courier).

After this period, a 3 mm-diameter disposable biopsy punch (Integra Miltex, NY, USA) was used to cut out the spots from the cards. The two discs for each sample, plus 70 µl of dilution buffer (2% BSA, 0.1% Tween-20 in PBS 1X), were then placed in a well in a 96-well plate and incubated at room temperature for 1 h in a shaker to elute all cytokines and chemokines from the spots. The concentrations of eluted IFN-γ, IL-2, CXCL10, CXCL9, CCL2 and CXCL8 in the DPS-FZ and DPS-AT were then determined as above.

### Experiment 2: Field experiment

Blood was collected as above from healthy subjects resident in Mymensingh (Bangladesh) in October 2016, a resource-limited area endemic for *L. donovani*. Of these subjects, 12 were classified as asymptomatic (based on positive rK39 and DAT tests), and 13 as non-infected (negative controls). As above, 500 µL of these blood samples were left either untreated (control) or were SLA-stimulated for 24 h^6^. The naturally resulting plasma fraction was then divided into two aliquots. The first was frozen at −20 °C, and the second was dropped on separate Protein Saver 903 cards as above to provide similar DPS-FZ and DPS-AT samples. The SLA-stimulated plasma (frozen at −20°C) and the DPS-FZ (−20 °C) and DPS-AT (ambient temperature) samples were sent by courier to our laboratory in Spain for analysis. This involved a 10 day ‘preparation-to pick-up-to-reception’ delivery period.

### Statistical analyses

Cytokine and chemokine concentrations were compared using the Mann-Whitney U test. Pearson correlation coefficients were calculated between the TLP, and DPS-FZ and DPS-AT treatments. The ability and cut-off point of the cytokines/chemokines to detect asymptomatic participants was determined by calculating the area under the receiver operating characteristic curve (AUC) plus the 95% confidence interval. Significance was set at P < 0.05. Analyses were performed GraphPad Prism 7.0 (GraphPad Software) and SPSS software (IBM SPSS Statistic 22).

### Data availability

The data sets generated during the current study are available on request.

## Results

### Proof of concept experiment

#### Cytokines/chemokines in the thawed liquid plasma samples and dried plasma spots: samples from *L. infantum*-endemic area

**Figure 1:**
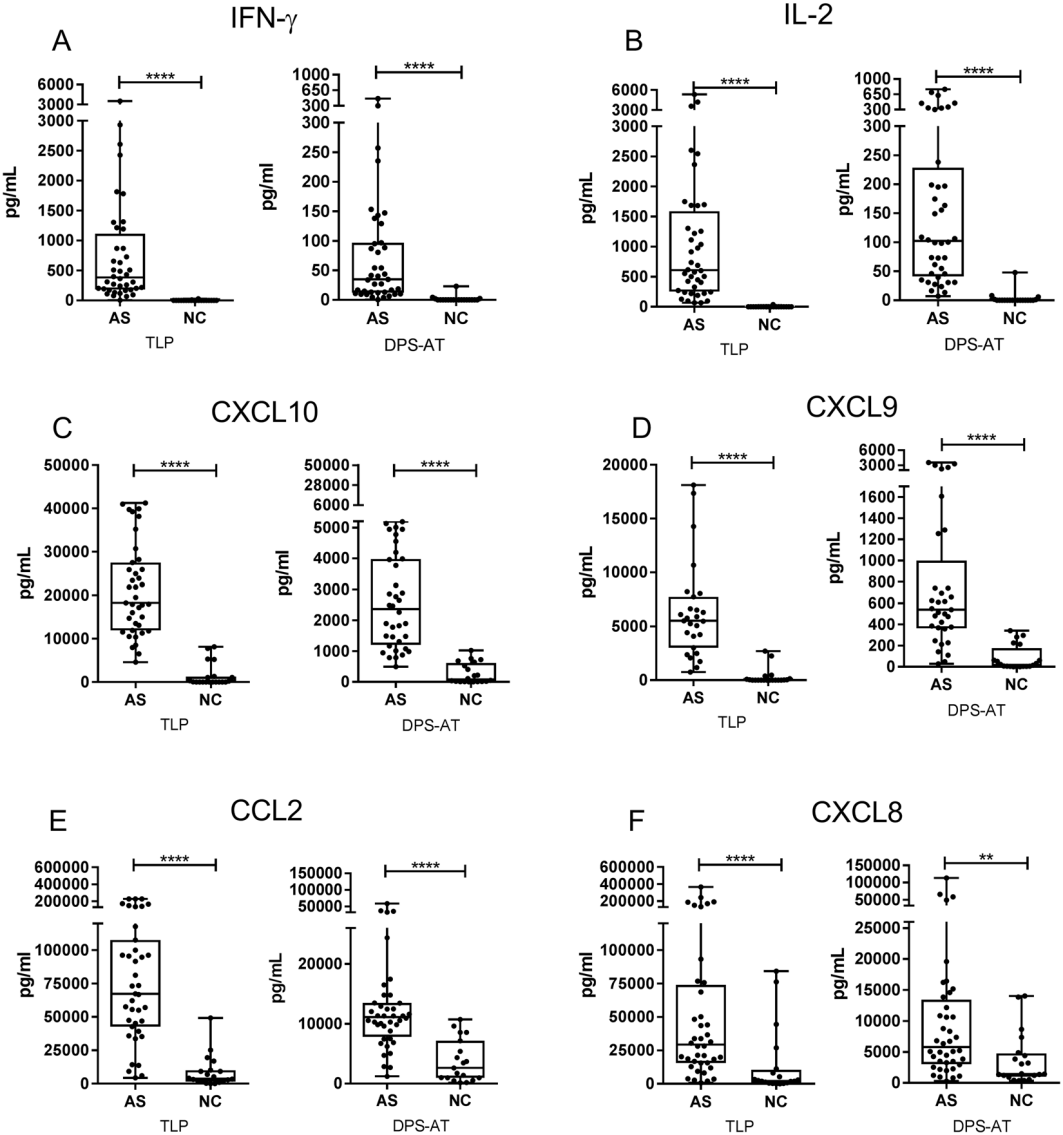
Concentrations of IFN-γ (**A**), IL-2 (**B**), CXCL10 (**C**), CXCL9 (**D**), CCL2 (**E**) and CXCL8 (**F**) in the TLP and DPS-AT treatments (DPS-FZ results not shown given their similarity to the DPS-AT results), for asymptomatic individuals (AS) and negative control (NC) subjects from the *L. infantum*-endemic area. Each dot represents one individual; box-whisker plots show median, interquartile range and min/max values. The Mann-Whitney U test was used to compare means. **p < 0.01; ****p < 0.0001. TLP - thawed liquid plasma; DPS-AT - dried plasma spot at ambient temperature.

**Figure 2:**
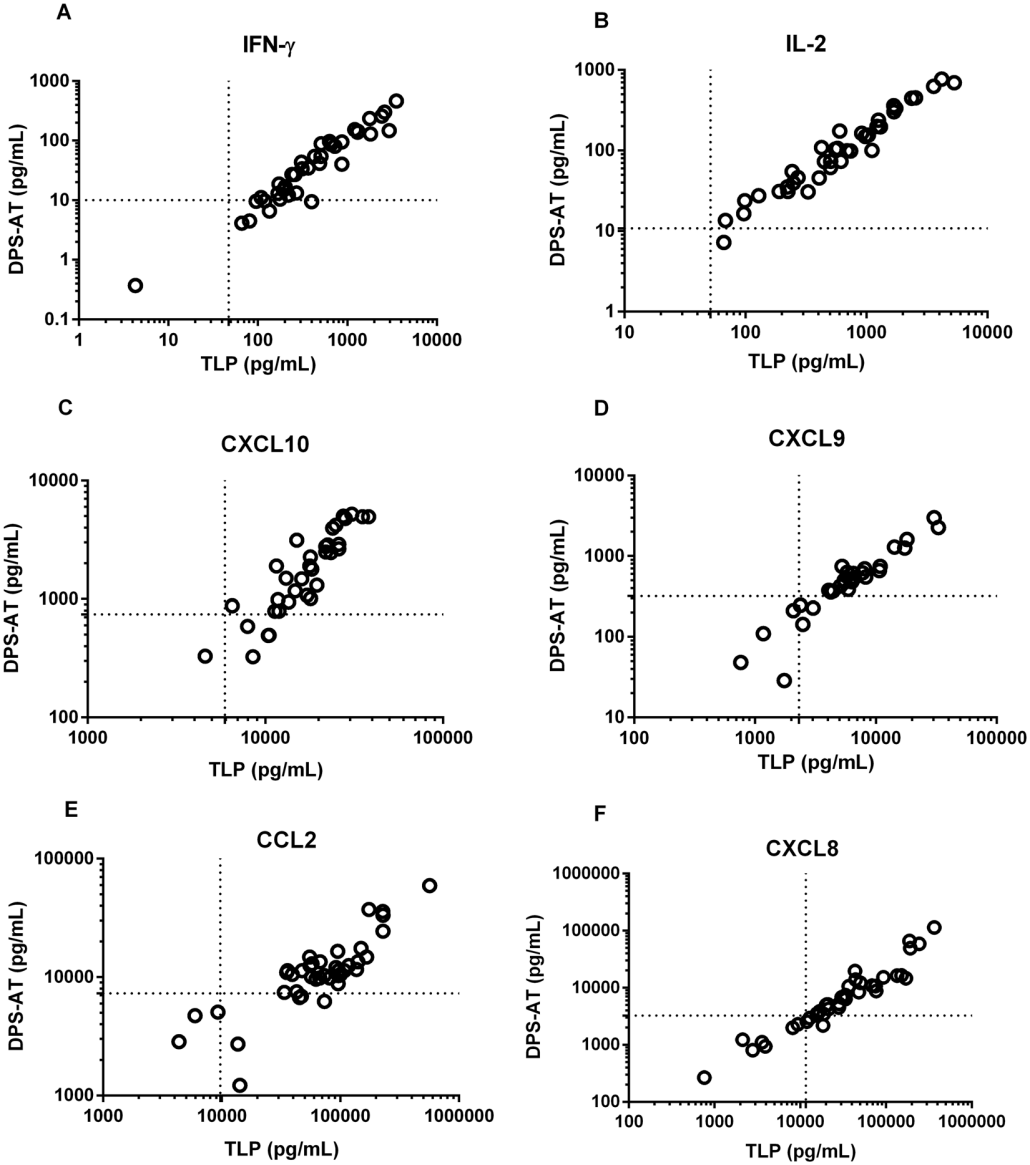
Correlation plots between cytokine/chemokine concentrations in TLP and DPS-AT samples (which are also representative of DPS-FZ samples) from asymptomatic subjects from the *L. infantum*-endemic area. (**A**) IFN-γ, (**B**) IL-2, (**C**) CXCL10, (**D**) CXCL9, (**E**) CCL2 and (**F**) CXCL8 for all asymptomatic subjects (n = 40). Lines denote cut-offs for positive tests. TLP - thawed liquid plasma; DPS-AT - dried plasma spot at ambient temperature.

As expected, in the TLP samples the cytokine and chemokine concentrations recorded were higher in the asymptomatic subjects than in the negative controls (p < 0.0001) (Fig. 1), and 6–10 times higher than those recorded for the corresponding DPS-FZ and DPS-AT samples. Differences were, however, also detectable between asymptomatic subjects and negative controls in both the DPS-FZ and DPS-AT samples with respect to all the cytokines and chemokines examined (p < 0.01 and p < 0.0001). Very strong correlations were detected between the TLP samples and the DPS-FZ and DPS-AT samples for all cytokine and chemokines (Fig. 2 and Supplementary Table 1).

### Field Experiment

#### Cytokines/chemokines in the thawed liquid plasma samples and dried plasma spots: samples from *L. donovani*-endemic area

Strong correlations were again seen between the TLP and DPS-FZ results, and the TLP and DPS-AT results, for all cytokines and chemokines (Supplementary Table 2). Again, the concentrations measured in the TLP samples from asymptomatic subjects were 6–10 times higher than those recorded in the DPS-FZ/AT spots (Supplementary Table 3).

**Figure 3:**
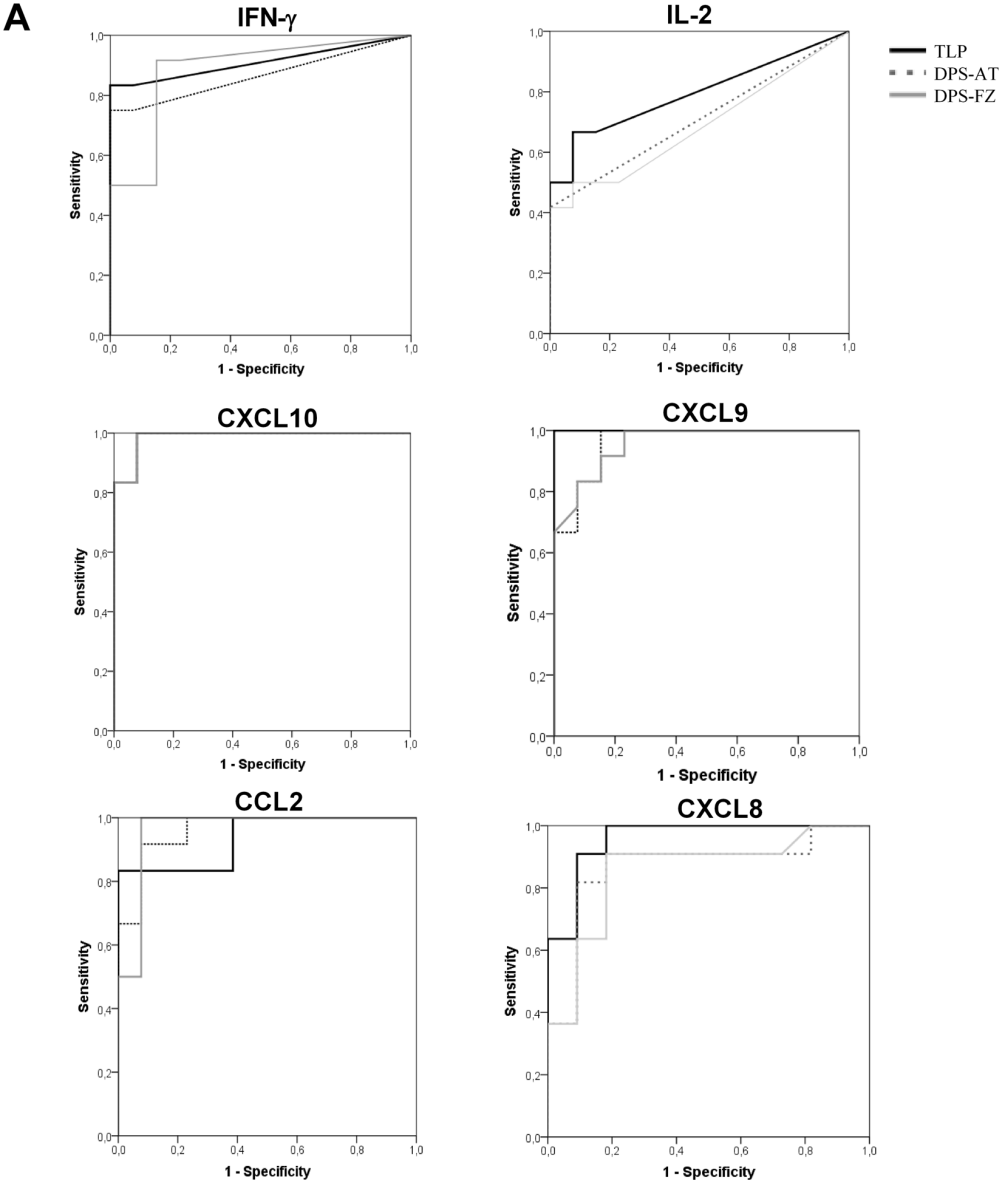
Receiver operating characteristic curve analysis for IFN-γ, IL-2, CXCL10, CXCL9, CCL2 and CXCL8 in thawed liquid plasma (TLP), and dried plasma spots maintained at ambient temperature (DPS-AT) or at −20 °C (DPS-FZ), prepared from blood extracted from subjects exposed to *L. donovani* in Bangladesh.

The AUCs for the TLP, DPS-FZ and DPS-AT treatments showed no significant differences with respect to any of the measured cytokines/chemokines, except for IL-2 (Fig. 3). Cytokines expressed at low levels, such as IFN-γ and IL-2, showed lower sensitivity values for the DPS-FZ/AT samples than for the TLP samples. However, the more strongly expressed chemokines CXCL10, CXCL9, CCL2 and CXCL8 showed similar values under all treatment conditions (Table 1).

**Table 1:** AUC, sensitivity and specificity values for IFN-γ, IL-2, CXCL10, CXCL9, CCL2 and CXCL8 in TLP, DPS-AT or DPS-FZ from SLA-stimulated plasma from asymptomatic *L. donovani* infected individuals.

**Analytes**	**TLP**				**DPS-RT**				**DPS-FZ**			
	AUC	p value	Se (%)	Sp (%)	AUC	p value	Se (%)	Sp (%)	AUC	p value	Se (%)	Sp (%)
**IFN-γ**	0.91	0.0005	83.33	92.31	0.87	0.0019	25.00	100	0.89	0.0011	25.00	100
**IL-2**	0.79	0.0123	66.67	92.31	0.71	0.0771	25.00	100	0.70	0.1027	33.33	100
**CXCL10**	0.99	<0.0001	83.33	92.31	0.99	<0.0001	83.33	92.31	0.99	<0.0001	83.33	92.31
**CXCL9**	1.00	<0.0001	100	100	0.96	<0.0001	83.33	92.31	0.96	<0.0001	83.33	92.31
**CCL2**	0.97	<0.0001	91.67	100	0.96	<0.0001	91.67	92.31	0.96	<0.0001	91.67	92.31
**CXCL8**	0.96	0.0003	90.91	90.91	0.87	0.0035	81.82	90.91	0.86	0.0047	90.91	81.82

TLP: thawed liquid plasma; DPS-RT: dried plasma spot at ambient temperature; DPS-FZ: dried plasma spot at −20 °C; AUC: area under the curve; Se: sensitivity; Sp: specificity.

## Discussion

The World Health Organization has recently suggested the use of SLA-stimulated blood as a means of screening for leishmaniasis infection in healthy populations^21^. In remote and resource-poor settings, however, the facilities required for maintaining plasma samples frozen until analysis are commonly lacking. Dried plasma spots provide a potentially useful and inexpensive means of overcoming this problem. Samples can be easily and quickly collected on filter paper cards and shipped at ambient temperature by post to an appropriate laboratory. The present work examines the value of Protein Saver 903 cards for transporting SLA-stimulated plasma samples as dried spots held at −20 °C and at ambient temperature. The results show they provide an excellent alternative to transporting larger frozen plasma samples for the later quantification of cytokine/chemokines. Indeed, the results of the field experiment performed in a village in Bangladesh confirm this method provides reliable plasma samples for later analysis, even when these are sent at ambient temperature.

The cytokines IL-2 and IFN-γ, and the chemokines CXCL10, CXCL9, CCL2 and CXCL8 were easily eluted from the dried spots on the cards. Although the concentrations recorded in the elutes were lower than those recorded in the TLP samples, they were equally able to identify asymptomatic subjects in *L. infantum* and *L. donovani* areas.

Previous studies undertaken by our group have shown that IL-2 from SLA-stimulated plasma to be 100% sensitive and specific for the detection of asymptomatic subjects in a *L. infantum*-endemic area^6^. CXCL10 and CXCL9 were also identified as robust biomarkers for detecting asymptomatic individuals in both *L. infantum-* and *L. donovani-*endemic areas, with high sensitivity and specificity^20^. This work consolidates the idea that CXCL10 and CXCL9 can be used as biomarkers of asymptomatic subjects, and shows that both these chemokines remain stable on filter paper at ambient temperature for at least 10 days.

The ability of this method to measure the cellular immune response against *Leishmania* should also be analysed in patients under treatment for visceral leishmaniasis. CXCL10, CXCL9 and IFN-γ have been proposed as potential biomarkers of cure^20^ and, as shown by the present results, they remain stable in DPS. Further, the present paper-carriage technology has been widely used in the serological and molecular identification of *Leishmania* ^14,22^. DBS have also been used for monitoring HIV viral loads in cytomegalovirus^13^, tuberculosis^23^ and *Leishmania* co-infections^24^. The present cards might also be used follow the cellular immune response of patients with HIV/*Leishmania* in treatment trials^12^. Recently, DPS samples have been described to provide a valid alternative to plasma sampling for the quantification of miltefosine in remote regions^25^. The ability to easily monitor the plasma concentration of the drug is of interest in large multicentre studies.

Because of its simplicity and portability, DPS collection could be widely used in remote areas, provided that the filter papers can be sent to an appropriate laboratory. The present plasma spots remained stable for 10 days at ambient temperature. Fully automated high-throughput card punching and analysis systems are routinely used in neonatal screening, and could be adapted for measuring any of the present cytokines/chemokines in a central laboratory^26^.

The small sample size of present study, however, means further validation of the DPS-AT should be undertaken with a larger number of subjects. It may also be useful to determine whether the results obtained are reproducible after longer courier transport times and when samples are exposed to different ambient temperatures. Even in the same remote area, the temperature may fluctuate widely between one time of year and another. Ambient temperature conditions may also vary between different forms of transport, which might affect how long the different cytokines/chemokines remain stable for reliable analysis.

In conclusion, this work establishes the proof of concept that Protein Saver 903 cards provide an alternative method for transporting SLA-stimulated plasma samples, even at ambient temperature, for the later analysis of IL-2, IFN-γ, CXCL10, CXCL9, CCL2 and CXCL8 concentrations. CXCL10 and CXCL9 DPS were found to be robust biomarkers for the identification of asymptomatic subjects living in *L. infantum*- and *L. donovani*-endemic areas. The DPS method could increase access to samples and reduce the costs of epidemiological and outbreak studies in remote settings. This would help in the control of *Leishmania* infection, and perhaps facilitate the running of clinical trials.

## Electronic supplementary material

Supplementary Tables
