# Supplementary material for: Cytokines and chemokines measured in dried SLA-stimulated whole blood spots for asymptomatic Leishmania infantum and Leishmania donovani infection
Source: Sci Rep. 2017 Dec 8;7:17266. doi: 10.1038/s41598-017-17315-z (PMC5722824; doi:10.1038/s41598-017-17315-z)
Supplement: Supplementary file 1 — Supplementary Tables [file 41598_2017_17315_MOESM1_ESM.doc]

**Supplementary information**

**Cytokines and chemokines measured in dried SLA-stimulated whole blood spots for asymptomatic *Leishmania* *infantum and Leishmania donovani* infection**

A.V. Ibarra-Meneses^1^, D. Mondal^2^, J. Alvar^3^, J. Moreno^1^ and E. Carrillo^1^*

^1^ WHO Collaborating Centre for Leishmaniasis, Centro Nacional de Microbiología, Instituto de Salud Carlos III, Madrid, Spain.

^2^ Nutrition and Clinical Services Division (NCSD), International Centre for Diarrhoeal Disease Research, Dhaka, Bangladesh.

^3^Drugs for Neglected Diseases Initiative (DNDi), Geneva, Switzerland.

*Correspondence to ecarrillo@isciii.es

**Supplementary Material**

**Supplementary Table 1.** Pearson correlation coefficients between TLP and DPS-AT/FZ results for asymptomatic subjects from the *L. infantum*-endemic area. We observed very strong correlations between the TLP samples and the DPS-FZ and DPS-AT samples for all cytokines and chemokines.

| **Analytes** | **WBA SLA *vs* DPS-AT** | **p value*** | **WBA SLA *vs* DPS-FZ** | **p value*** |
| --- | --- | --- | --- | --- |
| **IFN-γ** | 0.9306 | <0.0001 | 0.9460 | <0.0001 |
| **IL-2** | 0.9728 | <0.0001 | 0.9787 | <0.0001 |
| **CXCL10** | 0.9016 | <0.0001 | 0.9132 | <0.0001 |
| **CXCL9** | 0.9678 | <0.0001 | 0.9514 | <0.0001 |
| **CCL2** | 0.9185 | <0.0001 | 0.9291 | <0.0001 |
| **CXCL8** | 0.9253 | <0.0001 | 0.9390 | <0.0001 |

WBA: Whole blood assay; DPS-RT: dried plasma spot at ambient temperature; DPS-FZ: dried plasma spot frozen; *correlation coefficients were calculated by Pearson r.

**Supplementary Table 2.** Pearson correlation coefficients between TLP and DPS-AT/FZ results for asymptomatic subjects from the *L. donovani*-endemic area. Strong correlations were observed between the TLP samples and the DPS-FZ and DPS-AT samples for all cytokine and chemokine.

| **Analytes** | **WBA SLA *vs* DPS AT** | **p value*** | **WBA SLA *vs* DPS-FZ** | **p value*** |
| --- | --- | --- | --- | --- |
| **IFN-γ** | 0.8703 | <0.0001 | 0.8962 | <0.0001 |
| **IL-2** | 0.7948 | <0.0001 | 0.8207 | <0.0001 |
| **CXCL10** | 0.9422 | <0.0001 | 0.9529 | <0.0001 |
| **CXCL9** | 0.8761 | <0.0001 | 0.8845 | <0.0001 |
| **CCL2** | 0.8984 | <0.0001 | 0.9214 | <0.0001 |
| **CXCL8** | 0.9176 | <0.0001 | 0.9190 | <0.0001 |

WBA: Whole blood assay; DPS-RT: dried plasma spot at ambient temperature; DPS-FZ: dried plasma spot frozen; *correlation coefficients were calculated by Pearson r.

**Supplementary Table 3.** Median concentrations of cytokine/chemokine measured in the TLP, DPS-FZ and DPS-AT spots in asymptomatic subjects living in the *L. donovani*-endemic area. The concentration of the cytokines and chemokines in TLP samples from asymptomatic subjects were 6-10 times higher than those recorded in the DPS-FZ/AT spots.

| Analytes | **TLP** | | | **DPS-AT** | | | **DPS-FZ** | | |
| --- | --- | --- | --- | --- | --- | --- | --- | --- | --- |
|  | AS (pg/mL) | NC  (pg/mL) | p value | AS (pg/mL) | NC (pg/mL) | p value | AS  (pg/mL) | NC  (pg/mL) | p value |
| **IFN-γ** | 187.87 | 0.00 | <0.0001 | 1.92 | 0.00 | 0.0002 | 2.94 | 0.00 | 0.0003 |
| **IL-2** | 77.32 | 0.00 | 0.0039 | 0.00 | 0.00 | 0.0149 | 3.13 | 0.00 | 0.0719 |
| **CXCL10** | 16,040 | 167.59 | <0.0001 | 1435 | 59.11 | <0.0001 | 1416 | 63.30 | <0.0001 |
| **CXCL9** | 3598 | 58.28 | <0.0001 | 205.34 | 10.56 | <0.0001 | 241.56 | 12.50 | <0.0001 |
| **CCL2** | 21,521 | 1491 | <0.0001 | 2134 | 432.15 | <0.0001 | 2212 | 618.90 | <0.0001 |
| **CXCL8** | 50,477 | 4980 | <0.0001 | 9316 | 2264 | 0.0024 | 9105 | 2243 | 0.0033 |

TLP: thawed liquid plasma; DPS-RT: dried plasma spot at ambient temperature; DPS-FZ: dried plasma at -20ºC; AS: asymptomatic individuals; NC: negative control
